# 2-For-1 offer: *Bradysia polonica* (Lengersdorf, 1929) and *Bradysia spinidensa* Hondru, 1968, stat. res. (Diptera, Sciaridae)

**DOI:** 10.3897/BDJ.13.e171689

**Published:** 2025-12-26

**Authors:** Roy J Canty, Hans-Georg Rudzinski, Dominic Wanke, Daniel Whitmore

**Affiliations:** 1 State Museum of Natural History Stuttgart, Rosenstein 1, D-70191, Stuttgart, Germany State Museum of Natural History Stuttgart, Rosenstein 1, D-70191 Stuttgart Germany; 2 Center of Excellence for Biodiversity and Integrative Taxonomy (KomBioTa), Wollgrasweg 23, D-70599, Stuttgart, Germany Center of Excellence for Biodiversity and Integrative Taxonomy (KomBioTa), Wollgrasweg 23, D-70599 Stuttgart Germany; 3 University of Hohenheim, Garbenstraße 30, 70599, Stuttgart, Germany University of Hohenheim, Garbenstraße 30, 70599 Stuttgart Germany; 4 Breslauer Str. 8/b, D-28790, Schwanewede, Germany Breslauer Str. 8/b, D-28790 Schwanewede Germany

## Abstract

**Background:**

During the identification and sorting of German Sciaridae as part of the GBOL III “Dark Taxa” project, it was noted that specimens identified as *Bradysia polonica* (Lengersdorf, 1929) displayed such morphological variation that they could potentially be split into at least two morphospecies. An integrative taxonomic approach, utilising molecular and morphological data, confirmed the presence of two species, with one species being identifiable as *Bradysia spinidensa* Hondru, 1968, a species previously synonymised under *B. polonica*.

**New information:**

Using an integrative taxonomic approach combining molecular and morphological data, the observed morphological variation of *Bradysia polonica* (Lengersdorf, 1929) was investigated. The molecular data combined with the morphology of the gonostyli confirmed the presence of two species, with *Bradysia spinidensa* Hondru, 1968 being reinstated as a valid species.

## Introduction

The Sciaridae are one of the most diverse Diptera families, and are typically small, morphologically uniform, drably coloured, and inconspicuous, making them a challenge to identify (Mohrig et al. 2013, Du et al. 2024, Gorban et al. 2024). They are, therefore, often referred to as a “dark taxon”: a species-rich group that conceals many hidden species, is taxonomically challenging, is often understudied, and is worked on by a small number of taxonomists in regards to the taxon size (Hausmann et al. 2020, Chimeno et al. 2022).

This made the family a key candidate for the German Barcode of Life (GBOL) III “Dark Taxa” project (Hausmann et al. 2020). This project continued the previous two phases of the German Barcode of Life initiative, which was launched in 2011, and was specifically focused on the study of “dark taxa”, including the Sciaridae. The main aims of the GBOL III: “Dark Taxa” project are to enhance the size and quality of the German DNA barcode reference library, utilise an integrative taxonomic approach to study “Dark Taxa”, and to train a new generation of taxonomists (Hausmann et al. 2020). This study came from a subproject in GBOL III focusing on the Sciaridae, based at the Staatliches Museum für Naturkunde Stuttgart (SMNS).

In recent years, advancements have been made in the use of molecular data for species delimitation in organismal biology (Leliaert et al. 2009, Leliaert et al. 2014), ASAP (Puillandre et al. 2020) being a recent method which has proven robust in studies (e.g. Girard et al. 2022, Macher et al. 2022, Awad et al. 2023, Oliveira et al. 2023). In Sciaridae studies, integrating the use of COI barcodes with morphology has proved helpful not only for species delimitation (Shin et al. 2013, Shin et al. 2014, Katumanyane et al. 2020) but also for species discovery, and the reconstruction of phylogenetic relationships (Heller et al. 2016, Yang et al. 2019, Arthofer et al. 2021, Du et al. 2024, Shah et al. 2024).

With more than 450 described species, the genus *Bradysia* Winnertz, 1867 is one of the most speciose sciarid genera, and one of the most difficult to work with due to the high diversity, homogeneity, and likely polyphyletic origin of the genus (Yang et al. 2019, Du et al. 2024). Most of the Palearctic *Bradysia* species are ordered into 16 species groups (Menzel and Mohrig 2000). The *Bradysia polonica* group is one such group, proposed by Rudzinski (1996), containing the following species: *Bradysia malitiosa* Rudzinski, 1996, *Bradysia polonica* (Lengersdorf, 1929), *Bradysia pseudopolonica* Mohrig & Röschmann, 1994, and *Bradysia rubrascuta* Mohrig & Mamaev, 1989.

During the sorting of sciarid material by the authors for the GBOL III “Dark Taxa” project, it was noted that specimens preliminarily identified as *B. polonica* displayed some obvious morphological variation, especially in the gonostyli, potentially splitting them into at least two morphospecies. This raised the question of whether *B. polonica* is more than one species, and whether these divergent specimens belonged to a new species or if any of its synonyms should be reinstated as valid.

*Bradysia polonica* has two junior subjective synonyms: *Bradysia edwardsi* Freeman, 1983 and *Bradysia spinidensa* Hondru, 1968, synonymised by Menzel et al. (1990) and Menzel and Mohrig (2000), respectively, each without a written explanation. The holotype of *B. edwardsi* is deposited in the Natural History Museum, London (NHM), with images available online (NHM Data Portal 2024), with this specimen morphologically conforming to typical *B. polonica*. On the other hand, the situation with *B. spinidensa* is more complicated. In the original description, Hondru (1968) never mentioned where he deposited the types. Menzel and Mohrig (2000) specified that they were unable to locate the type material of *B. spinidensa* in Romania. They noted that they had no response to written requests to Hondru’s place of work due to the political and economic situation in Romania at the time, but they never named this location. When comparing our morphologically divergent specimens with the admittedly Spartan original description of *B. spinidensa* (Hondru 1968), they fit well with that nominal species.

In this study, we integrated molecular and morphological data to explore the “*B. polonica*” sample found during the project, as well as specimens made available to us by the Museum Koenig Bonn, along with their respective COI barcode data. We analysed the COI data using ASAP to delimit potential molecular species clusters, which were then compared with a more robust phylogenetic tree for congruence. The molecular data was then compared with the morphology to determine how many and which species were present in the sample.

## Materials and methods

### Sorting and specimen selection

As part of the broader efforts to produce DNA barcode sequences of German Sciaridae within the GBOL III “Dark Taxa” project, 7964 specimens were sourced from samples collected throughout the various phases of the GBOL initiative. As the male genitalia are typically used for morphological identifications in Sciaridae (Mohrig and Menzel 2009, Broadley et al. 2016), and this is where the clearest morphological differences could be observed, male specimens were prioritised during this study. The specimens were initially sorted to genus level at the SMNS using a Leica M125 C stereo microscope, with some of these specimens (2621 specimens) being sent to National University of Singapore (NUS) for nanopore sequencing following the LIT method (Large-Scale Integrative Taxonomy; see Hartop et al. 2022). Specimens sorted after this first round of sequencing were processed for DNA extractions and sequencing, mainly in-house at the SMNS. Further details of the DNA extraction and sequencing processes are outlined below. Species-level identification, along with training in sciarid identifications, was undertaken at the home of Hans-Georg Rudzinski. This required briefly soaking specimens in lactic acid for dissection under a PZO 22052 stereo microscope, the production of temporary microscope slides, with a lactic acid medium, observation under a LEICA DM750 compound microscope. Temporary mounts were used, as they allow for the observation of structures in different positions, e.g., the ventral and dorsal aspects of the hypopygium. During the initial morphological identification of this material, it was noted that specimens roughly identifiable as *Bradysia polonica* exhibited morphological variation in the gonostyli, which suggested that we were dealing with at least two morphospecies.

### DNA extraction, amplification, and sequencing

Three series of DNA extraction, amplification, and sequencing were undertaken to produce 260 COI sequences for the target group of this study: an initial run with specimens sent to the NUS, producing 119 sequences for the target species-group; in-house (at the SMNS) DNA extraction, and amplification for Sanger sequencing (off-site), producing 139 sequences for the target species-group; and in-house DNA extraction, amplification, and sequencing using the MinION, producing 2 sequences for the target species-group. The methodologies for each follow thusly.

For specimens sent to the NUS, DNA extraction, PCR, and sequencing procedures largely followed the methodology outlined in Hartop et al. (2022). The “HotSHOT” methodology, as presented by Srivathsan et al. (2019), was used for non-destructive DNA extraction. Full length COI barcodes were then produced by PCR amplification using the following primers: LCO1490 (5'-GGTCAACAAATCATAAAGATATTGG-3'; Folmer et al. 1994) and modified jgHCO2198 (5′-TAIACYTCIGGRTGICCRAARAAYCA-3′; Geller et al. 2013). The primers were tagged as per the method first developed by Meier et al. (2015). Sequences were then obtained using a MinION (by Oxford Nanopore Technologies) equipped with an R10.3 flow-cell, as per Srivathsan et al. (2021). Following sequencing, basecalling using Guppy 2.3.5+53a111f, and demultiplexing using ONT Barcoder (Srivathsan et al. 2021: https://github.com/asrivathsan/ONTbarcoder), were conducted. The sequence reads obtained were then aligned using MAFFT v7 (Katoh and Standley 2013), and the MinION data was processed according to the bioinformatics pipeline outlined by Srivathsan et al. (2019).

In-house, two methods of non-destructive DNA extraction and two PCR methods were used, depending on availability. The first extraction method, used in conjunction with Sanger sequencing, was automated extraction using a Xiril Neon 100 pipetting robot (SMNS) following the protocol outlined in Haas et al. (2021), apart from a non-destructive extraction technique (only soaking the specimens in the buffer solution) rather than the semi-destructive technique suggested by the protocol. The second extraction method, used in conjunction with both Sanger and MinION sequencing, was the “HotSHOT” methodology outlined in Srivathsan et al. (2021), but modified for a 20 μl buffer solution (10 μl alkaline buffer + 10 μl neutralising buffer) and a 20-minute extraction run (18 minutes at 65 °C and 2 minutes at 98 °C). PCR amplification for both Sanger and MinION sequencing utilised 25 μl of reaction mix with 4 μl of DNA template, and followed the cycler protocol of Haas et al. (2021). Two different sets of primers were used for the PCR, depending on the sequencing method to be used afterwards. For Sanger sequencing, the following primers were used: LCO1490, and HCO 2198 (5′-TAAACTTCAGGGTGACCAAAAAATCA-3′) (Folmer et al. 1994). For MinION sequencing, the primers used were the same as those used at NUS (LCO1490 from Folmer et al. (1994), modified jgHCO2198 from Geller et al. (2013)).

For Sanger sequencing, DNA extracts were sent to a Eurofins Genomics laboratory operating in the University of Hohenheim, Stuttgart, Germany for bidirectional sequencing. MinION sequencing, on the other hand, was performed in-house and followed the protocol of Srivathsan et al. (2021) without modification, to produce full-length COI barcodes. The sequences obtained were then aligned and inspected in-house using Geneious Prime 2023.2.1 (https://www.geneious.com).

In addition to these newly-obtained COI sequences, 29 additional COI sequences, along with their respective specimens (27 identified as *B. polonica*; 2 identified as *B. edwardsi*), were received from the collections of The Zoologisches Forschungsmuseum Alexander Koenig, Bonn (ZFMK) for both the molecular and morphological analyses. These specimens are labelled with ZFMK and GBOL numbers, with the DNA extraction, amplification, and sequencing methods following the protocol outlined in Müller et al. (2024). All specimens used for molecular analyses are accessible in BOLD (Barcode of Life Datasystems) in the public dataset DS-Bradysia (doi: dx.doi.org/10.5883/DS-BRADYSIA).

### Molecular analyses

Molecular data for *B. polonica*-group were analysed following a two-step process: the building of partitions to delimit potential molecular species with ASAP, and the building of a phylogenetic tree (with an outgroup of *Schwenckfeldina carbonaria* (Meigen, 1830)) via IQ Tree, for comparison. The online version of ASAP (Puillandre et al. 2020), with the simple p-distances substitution model selected, was used for the building of partitions.

To produce the phylogenetic tree, it was necessary to first determine the correct substitution model. The ModelFinder application (Kalyaanamoorthy et al. 2017) on the IQ-Tree web server (Nguyen et al. 2014, Trifinopoulos et al. 2016) was utilised for this first step. For the phylogenetic analysis, the following substitution model and options were suggested and selected: the Hasegawa-Kishino-Yano substitution model (HKY: Hasegawa et al. 1985) with empirical state frequency (F) and Gamma rate heterogeneity with 4 rate categories (G4). Two different branch support analyses were run: SH-aLRT (Guindon et al. 2010) and ultrafast bootstrap 2 (UFBoot2; Hoang et al. 2018). The tree was then compared with the ASAP outputs to check for congruence. Molecular species groups coming out of the molecular delimitation were then used as hypothetical species clusters to compare with the morphology of the specimens, in order to determine congruence between molecular delimitation and morphological delimitation.

### Morphology

Specimens for observation were selected for comparison based on the best scoring (lowest ASAP-score, indicating the most likely molecular delimitations) molecular clusters from the ASAP results (Table 1), i.e. a subsample of 34 specimens from a larger cluster of 280 specimens (identified as *Bradysia polonica* in Fig. 2), and 6 specimens from a smaller cluster of 9 specimens (Identified as *Bradysia spinidensa* in Fig. 2). Specimens from within and between the two clusters were compared morphologically. Morphological terminology follows that of Menzel and Mohrig (2000) and Mohrig et al. (2013).

At the home laboratory of Hans-Georg Rudzinski, selected specimens were briefly soaked in lactic acid for dissection of the following structures under a PZO 22052 stereo microscope: hypopygium, wings, legs, head. Each dissected specimen was mounted onto a slide in lactic acid, for observation under a LEICA DM750 compound microscope. The main observations were made on the gonostyli (part of the hypopygium) as this is where the two species differ morphologically. Our observations included the number and arrangement of the two different types of spines (the thicker, darker spines, and thinner, hyaline spines), and measurements of the total length (L; from the apex to the base, before the basal lobe) and breadth at mid length (mB) of the gonostyli to produce an L/mB index.

### Imaging

Photographs were taken with a Zeiss Axiocam 503 color camera connected to a Zeiss Axio Imager.M2 microscope, in conjunction with the ZEN pro 3.4 microscopy software by Zeiss Microscopy and utilising the z-stacking option to produce a series of images through one vertical plane covering the full depth of the body part of interest. Each set of images was then concatenated and edited into one full depth image in Helicon Focus 8.1.0 to produce the final image.

## Taxon treatments

### Bradysia polonica

0BCA08DE-798F-5D00-9511-F8545ABDF743

#### Description

Containing: *Bradysia malitiosa* Rudzinski, 1996, *Bradysia polonica* (Lengersdorf, 1929), *Bradysia pseudopolonica* Mohrig & Röschmann, 1994, and *Bradysia rubrascuta* Mohrig & Mamaev, 1989 (Rudzinski 1996).

According to Menzel and Mohrig (2000), species in the *B. polonica* group are characterised by the following morphological character states: Antenna base uniformly dark; basal parts rough (without transversely wrinkled surface structure and without sensilla); neck parts uniformly coloured; sensilla field on the basal palpal segment simple and without borders (only sometimes finely bordered); 2nd palpal segment with a distinct, somewhat elongated outer bristle; abdomen finely and sparsely haired; scutellum with 2 strong, long marginal bristles; mesonotum finely and sparsely haired, strong marginal bristles present; fore tibia with spines among the basal bristles; inner-apex of fore-tibia with single-row bristle comb and coarse-bristled spot; single-row bristle comb narrow and with a basal ridge (at most 1/2 times as wide as the width of the tibia tip); claws untoothed; vein R_1_ short, converging into vein C, clearly or far before the m-fork base; vein R_5_ apically with one-sided dorsal macrotrichia (rarely also with 1 to 3 ventral macrotrichia in the anterior half); ventral inner sides of valves of hypopygium V-shaped, emarginated; ventral genital base with a central basal lobe (this is relatively broad, only covered with strong marginal bristles and strongly sclerotized); basal lobe arising close to 9th tergite and overlapping the inner edge of the valves; inner edges of valves relatively short-haired; stylus compact and clearly thickened; stylus tip roundish and without a tooth (at most subapically with 1 to 2 strong, tooth-shaped curved spines on upright high bases); tip hairs of styli coarse and sparse; inner side of stylus quite deeply emarginated to the middle; ventral stylus margin with several strong (often additionally with fine-hyaline and sometimes also long curved) spines; genital plate broad and weakly sclerotised; tip of genital plate broadly rounded; basal processes relatively short and not noticeably thickened; dentate field small and circular, with rather fine and single-pointed teeth; aedeagus short and slender, with a weak, short-conical base.

### Bradysia polonica

(Lengersdorf, 1929)

F5177275-A36B-509B-8215-1844A56E3ACB

(Sciara (Neosciara) polonica Lengersdorf 1929 - Bull. Acad. Pol. Sci. (Zool.), 1929 (3–4): 110-111; 112, Fig. 2). Type locality: Galizia (Poland). Lectotype: 1 male, "Wiklina" (= willow), 6.VII.1869, leg. GRZEGORZEK. Paralectotypes: 3 females, “Wiklina" (= willow), 30.VI.1869, 10.VII.1869, 20.VII.1869, leg. GRZEGORZEK. Type depository: Lectotype and Paralectotypes deposited in the collection of The Zoologisches Forschungsmuseum Alexander Koenig, Bonn (ZFMK).
= ***Bradysia edwardsi* Freeman, 1983** (synonymised by Menzel et al. (1990)). Freeman 1983 Entomologist's mon. Mag., 119: 166-167; Fig. 10. Type locality: Oxford, Bagley Wood, Great Britain (GB). Holotype: 1 male, 20.V.–21.V.1933, leg. EDWARDS. Paratypes: 2 males, Oxford, Bagley Wood, GB, 20.V.–21.V.1933, leg. EDWARDS; 7 males, Herts., Welwyn, GB, 21–24.VII.1934 Leg. SMITH; 2 males, Letchworth, GB, 1 male VI.1917, 1 male IX.1917, leg. SMITH; 2 males, Hunts, Hemingford, GB, IX.1938, leg. SMITH; 1 male, Lancashire, Warrington, GB, 10.IV.35, leg. SMITH; 1 male, Berks., Cothill, GB, 26.V.1936, leg. EDWARDS; 2 males, N.E. Yorks, Mulgrave Woods, GB, 23.VIII–3.IX.1937, leg. EDWARDS; 1 male, Perthshire, Killin Distr., Ben Chalum, GB, 9–10.VI.1932, leg. EDWARDS. Type depository: Holotype and Paratypes are deposited in the collection of the Natural History Museum, London (NHM).

#### Materials

- **Type status:** Other material. **Occurrence:** catalogNumber: GBOL-224102203; recordedBy: M. Engelhardt, Ch. Koenig, T. Kothe; individualCount: 1; sex: male; lifeStage: adult; occurrenceID: BEA40D55-8A81-567E-9004-67A2495A4766; **Taxon:** scientificName: Bradysia polonica; **Location:** country: Germany; stateProvince: Baden-Württemberg; locality: Tübingen, Steinenberg; verbatimCoordinates: 48°31'51.6"N 9°1'51.6"E; **Identification:** identifiedBy: Kate Perez; **Event:** samplingProtocol: Malaise Trap; eventDate: 13/V/2014; **Record Level:** institutionID: ZFMK
- **Type status:** Other material. **Occurrence:** catalogNumber: GBOL-224102255; recordedBy: M. Engelhardt, Ch. Koenig, T. Kothe; individualCount: 1; sex: male; lifeStage: adult; occurrenceID: C1FDCB54-F030-513F-946D-3F42006BF19C; **Taxon:** scientificName: Bradysia polonica; **Location:** country: Germany; stateProvince: Baden-Württemberg; locality: Tübingen, Steinenberg; verbatimCoordinates: 48°31'51.6"N 9°1'51.6"E; **Identification:** identifiedBy: Kate Perez; **Event:** samplingProtocol: Malaise Trap; eventDate: 13/V/2014; **Record Level:** institutionID: ZFMK
- **Type status:** Other material. **Occurrence:** catalogNumber: SMNS_DIP_011311; recordedBy: F. Woog; individualCount: 1; sex: male; lifeStage: adult; occurrenceID: 53F90112-5579-52D0-A2FA-B3D186F9146B; **Taxon:** scientificName: Bradysia polonica; **Location:** country: Germany; stateProvince: Baden-Württemberg; locality: Stuttgart, Espan; verbatimElevation: 283 m; verbatimCoordinates: 48°48'49"N 9°15'01"E; **Identification:** identifiedBy: Roy J. Canty & Hans-Georg Rudzinski; **Event:** samplingProtocol: Malaise Trap; eventDate: 20/IX–20/X/2014; **Record Level:** institutionID: SMNS
- **Type status:** Other material. **Occurrence:** catalogNumber: SMNS_DIP_011337; recordedBy: A. Rosenbauer; individualCount: 1; sex: male; lifeStage: adult; occurrenceID: 10BBC1D6-2C31-57EE-B65D-196ECE73042A; **Taxon:** scientificName: Bradysia polonica; **Location:** country: Germany; stateProvince: Baden-Württemberg; locality: Backnang, Katharinenplaisir, Streuobstwiese; verbatimElevation: 294 m; verbatimCoordinates: 48°57'19.34"N 9°26'25.82"E; **Identification:** identifiedBy: Roy J. Canty & Hans-Georg Rudzinski; **Event:** samplingProtocol: Malaise Trap; eventDate: 20/VII–01/VIII/2014; **Record Level:** institutionID: SMNS
- **Type status:** Other material. **Occurrence:** catalogNumber: SMNS_DIP_011342; recordedBy: A. Rosenbauer; individualCount: 1; sex: male; lifeStage: adult; occurrenceID: 75512993-5F0A-572F-83E1-39F76408D1B4; **Taxon:** scientificName: Bradysia polonica; **Location:** country: Germany; stateProvince: Baden-Württemberg; locality: Backnang, Katharinenplaisir, Streuobstwiese; verbatimElevation: 294 m; verbatimCoordinates: 48°57'19.34"N 9°26'25.82"E; **Identification:** identifiedBy: Roy J. Canty & Hans-Georg Rudzinski; **Event:** samplingProtocol: Malaise Trap; eventDate: 20/VII–01/VIII/2014; **Record Level:** institutionID: SMNS
- **Type status:** Other material. **Occurrence:** catalogNumber: SMNS_DIP_011343; recordedBy: A. Rosenbauer; individualCount: 1; sex: male; lifeStage: adult; occurrenceID: 0105A604-A0C0-5E6B-BF14-96E6C498401B; **Taxon:** scientificName: Bradysia polonica; **Location:** country: Germany; stateProvince: Baden-Württemberg; locality: Backnang, Katharinenplaisir, Streuobstwiese; verbatimElevation: 294 m; verbatimCoordinates: 48°57'19.34"N 9°26'25.82"E; **Identification:** identifiedBy: Roy J. Canty & Hans-Georg Rudzinski; **Event:** samplingProtocol: Malaise Trap; eventDate: 20/VII–01/VIII/2014; **Record Level:** institutionID: SMNS
- **Type status:** Other material. **Occurrence:** catalogNumber: SMNS_DIP_011349; recordedBy: A. Rosenbauer; individualCount: 1; sex: male; lifeStage: adult; occurrenceID: BF0E12FB-D1DF-5178-8152-314F74384459; **Taxon:** scientificName: Bradysia polonica; **Location:** country: Germany; stateProvince: Baden-Württemberg; locality: Backnang, Katharinenplaisir, Streuobstwiese; verbatimElevation: 294 m; verbatimCoordinates: 48°57'19.34"N 9°26'25.82"E; **Identification:** identifiedBy: Roy J. Canty & Hans-Georg Rudzinski; **Event:** samplingProtocol: Malaise Trap; eventDate: 20/VII–01/VIII/2014; **Record Level:** institutionID: SMNS
- **Type status:** Other material. **Occurrence:** catalogNumber: SMNS_DIP_011350; recordedBy: A. Rosenbauer; individualCount: 1; sex: male; lifeStage: adult; occurrenceID: B0F19805-F2E5-5F2F-81E3-A26460F13576; **Taxon:** scientificName: Bradysia polonica; **Location:** country: Germany; stateProvince: Baden-Württemberg; locality: Backnang, Katharinenplaisir, Streuobstwiese; verbatimElevation: 294 m; verbatimCoordinates: 48°57'19.34"N 9°26'25.82"E; **Identification:** identifiedBy: Roy J. Canty & Hans-Georg Rudzinski; **Event:** samplingProtocol: Malaise Trap; eventDate: 20/VII–01/VIII/2014; **Record Level:** institutionID: SMNS
- **Type status:** Other material. **Occurrence:** catalogNumber: SMNS_DIP_011380; recordedBy: A. Rosenbauer; individualCount: 1; sex: male; lifeStage: adult; occurrenceID: 094B622C-C8CE-5D89-9557-FC6577B443FD; **Taxon:** scientificName: Bradysia polonica; **Location:** country: Germany; stateProvince: Baden-Württemberg; locality: Backnang, Katharinenplaisir, Streuobstwiese; verbatimElevation: 294 m; verbatimCoordinates: 48°57'19.34"N 9°26'25.82"E; **Identification:** identifiedBy: Roy J. Canty & Hans-Georg Rudzinski; **Event:** samplingProtocol: Malaise Trap; eventDate: 20/VII–01/VIII/2014; **Record Level:** institutionID: SMNS
- **Type status:** Other material. **Occurrence:** catalogNumber: SMNS_DIP_011383; recordedBy: A. Rosenbauer; individualCount: 1; sex: male; lifeStage: adult; occurrenceID: DA8798B2-11D6-52F7-BB1B-C3BB4E488C66; **Taxon:** scientificName: Bradysia polonica; **Location:** country: Germany; stateProvince: Baden-Württemberg; locality: Backnang, Katharinenplaisir, Streuobstwiese; verbatimElevation: 294 m; verbatimCoordinates: 48°57'19.34"N 9°26'25.82"E; **Identification:** identifiedBy: Roy J. Canty & Hans-Georg Rudzinski; **Event:** samplingProtocol: Malaise Trap; eventDate: 20/VII–01/VIII/2014; **Record Level:** institutionID: SMNS
- **Type status:** Other material. **Occurrence:** catalogNumber: SMNS_DIP_011393; recordedBy: A. Rosenbauer; individualCount: 1; sex: male; lifeStage: adult; occurrenceID: 4489AC57-8540-5029-9D34-D6305A172FB0; **Taxon:** scientificName: Bradysia polonica; **Location:** country: Germany; stateProvince: Baden-Württemberg; locality: Backnang, Katharinenplaisir, Streuobstwiese; verbatimElevation: 294 m; verbatimCoordinates: 48°57'19.34"N 9°26'25.82"E; **Identification:** identifiedBy: Roy J. Canty & Hans-Georg Rudzinski; **Event:** samplingProtocol: Malaise Trap; eventDate: 20/VII–01/VIII/2014; **Record Level:** institutionID: SMNS
- **Type status:** Other material. **Occurrence:** catalogNumber: SMNS_DIP_011398; recordedBy: A. Rosenbauer; individualCount: 1; sex: male; lifeStage: adult; occurrenceID: F4F91FF1-7F40-5104-8199-CA9BCE7C1242; **Taxon:** scientificName: Bradysia polonica; **Location:** country: Germany; stateProvince: Baden-Württemberg; locality: Backnang, Katharinenplaisir, Streuobstwiese; verbatimElevation: 294 m; verbatimCoordinates: 48°57'19.34"N 9°26'25.82"E; **Identification:** identifiedBy: Roy J. Canty & Hans-Georg Rudzinski; **Event:** samplingProtocol: Malaise Trap; eventDate: 20/VII–01/VIII/2014; **Record Level:** institutionID: SMNS
- **Type status:** Other material. **Occurrence:** catalogNumber: SMNS_DIP_011426; recordedBy: A. Rosenbauer; individualCount: 1; sex: male; lifeStage: adult; occurrenceID: B3B20B9D-C392-58DE-99C3-0CC32DE779DA; **Taxon:** scientificName: Bradysia polonica; **Location:** country: Germany; stateProvince: Baden-Württemberg; locality: Backnang, Katharinenplaisir, Streuobstwiese; verbatimElevation: 294 m; verbatimCoordinates: 48°57'19.34"N 9°26'25.82"E; **Identification:** identifiedBy: Roy J. Canty & Hans-Georg Rudzinski; **Event:** samplingProtocol: Malaise Trap; eventDate: 20/VII–01/VIII/2014; **Record Level:** institutionID: SMNS
- **Type status:** Other material. **Occurrence:** catalogNumber: SMNS_DIP_011427; recordedBy: A. Rosenbauer; individualCount: 1; sex: male; lifeStage: adult; occurrenceID: E6BAD608-D31A-59D5-894B-C8B3601735E6; **Taxon:** scientificName: Bradysia polonica; **Location:** country: Germany; stateProvince: Baden-Württemberg; locality: Backnang, Katharinenplaisir, Streuobstwiese; verbatimElevation: 294 m; verbatimCoordinates: 48°57'19.34"N 9°26'25.82"E; **Identification:** identifiedBy: Roy J. Canty & Hans-Georg Rudzinski; **Event:** samplingProtocol: Malaise Trap; eventDate: 20/VII–01/VIII/2014; **Record Level:** institutionID: SMNS
- **Type status:** Other material. **Occurrence:** catalogNumber: SMNS_DIP_011431; recordedBy: A. Rosenbauer; individualCount: 1; sex: male; lifeStage: adult; occurrenceID: 8D1F9031-7D73-54FC-B6DA-C90FCB490BDD; **Taxon:** scientificName: Bradysia polonica; **Location:** country: Germany; stateProvince: Baden-Württemberg; locality: Backnang, Katharinenplaisir, Streuobstwiese; verbatimElevation: 294 m; verbatimCoordinates: 48°57'19.34"N 9°26'25.82"E; **Identification:** identifiedBy: Roy J. Canty & Hans-Georg Rudzinski; **Event:** samplingProtocol: Malaise Trap; eventDate: 20/VII–01/VIII/2014; **Record Level:** institutionID: SMNS
- **Type status:** Other material. **Occurrence:** catalogNumber: SMNS_DIP_011435; recordedBy: A. Rosenbauer; individualCount: 1; sex: male; lifeStage: adult; occurrenceID: CD47EACF-2D53-51BC-8A8B-C1A95CA8A7AB; **Taxon:** scientificName: Bradysia polonica; **Location:** country: Germany; stateProvince: Baden-Württemberg; locality: Backnang, Katharinenplaisir, Streuobstwiese; verbatimElevation: 294 m; verbatimCoordinates: 48°57'19.34"N 9°26'25.82"E; **Identification:** identifiedBy: Roy J. Canty & Hans-Georg Rudzinski; **Event:** samplingProtocol: Malaise Trap; eventDate: 20/VII–01/VIII/2014; **Record Level:** institutionID: SMNS
- **Type status:** Other material. **Occurrence:** catalogNumber: SMNS_DIP_011455; recordedBy: A. Rosenbauer; individualCount: 1; sex: male; lifeStage: adult; occurrenceID: 9496A15F-8808-5E30-BE35-17F96C2756B1; **Taxon:** scientificName: Bradysia polonica; **Location:** country: Germany; stateProvince: Baden-Württemberg; locality: Backnang, Katharinenplaisir, Streuobstwiese; verbatimElevation: 294 m; verbatimCoordinates: 48°57'19.34"N 9°26'25.82"E; **Identification:** identifiedBy: Roy J. Canty & Hans-Georg Rudzinski; **Event:** samplingProtocol: Malaise Trap; eventDate: 20/VII–01/VIII/2014; **Record Level:** institutionID: SMNS
- **Type status:** Other material. **Occurrence:** catalogNumber: SMNS_DIP_022365; recordedBy: A. Rosenbauer; individualCount: 1; sex: male; lifeStage: adult; occurrenceID: 71370761-301F-52FA-8AE8-27BC13524E2A; **Taxon:** scientificName: Bradysia polonica; **Location:** country: Germany; stateProvince: Baden-Württemberg; locality: Backnang, Katharinenplaisir, Streuobstwiese; verbatimElevation: 294 m; verbatimCoordinates: 48°57'19.34"N 9°26'25.82"E; **Identification:** identifiedBy: Roy J. Canty & Hans-Georg Rudzinski; **Event:** samplingProtocol: Malaise Trap; eventDate: 28/V–16/VI/2014; **Record Level:** institutionID: SMNS
- **Type status:** Other material. **Occurrence:** catalogNumber: SMNS_DIP_022379; recordedBy: A. Rosenbauer; individualCount: 1; sex: male; lifeStage: adult; occurrenceID: A28A49DC-6CA3-562F-B58E-45734416C7B2; **Taxon:** scientificName: Bradysia polonica; **Location:** country: Germany; stateProvince: Baden-Württemberg; locality: Backnang, Katharinenplaisir, Streuobstwiese; verbatimElevation: 294 m; verbatimCoordinates: 48°57'19.34"N 9°26'25.82"E; **Identification:** identifiedBy: Roy J. Canty & Hans-Georg Rudzinski; **Event:** samplingProtocol: Malaise Trap; eventDate: 28/V–16/VI/2014; **Record Level:** institutionID: SMNS
- **Type status:** Other material. **Occurrence:** catalogNumber: SMNS_DIP_022383; recordedBy: A. Rosenbauer; individualCount: 1; sex: male; lifeStage: adult; occurrenceID: 962A9A19-6DE6-58D2-8431-42A52B6F55E9; **Taxon:** scientificName: Bradysia polonica; **Location:** country: Germany; stateProvince: Baden-Württemberg; locality: Backnang, Katharinenplaisir, Streuobstwiese; verbatimElevation: 294 m; verbatimCoordinates: 48°57'19.34"N 9°26'25.82"E; **Identification:** identifiedBy: Roy J. Canty & Hans-Georg Rudzinski; **Event:** samplingProtocol: Malaise Trap; eventDate: 28/V–16/VI/2014; **Record Level:** institutionID: SMNS
- **Type status:** Other material. **Occurrence:** catalogNumber: SMNS_DIP_022471; recordedBy: A. Rosenbauer; individualCount: 1; sex: male; lifeStage: adult; occurrenceID: 1E837B47-FEDD-5842-8F02-7D61CDD0A21D; **Taxon:** scientificName: Bradysia polonica; **Location:** country: Germany; stateProvince: Baden-Württemberg; locality: Backnang, Katharinenplaisir, Streuobstwiese; verbatimElevation: 294 m; verbatimCoordinates: 48°57'19.34"N 9°26'25.82"E; **Identification:** identifiedBy: Roy J. Canty & Hans-Georg Rudzinski; **Event:** samplingProtocol: Malaise Trap; eventDate: 28/V–16/VI/2014; **Record Level:** institutionID: SMNS
- **Type status:** Other material. **Occurrence:** catalogNumber: SMNS_DIP_022571; recordedBy: A. Rosenbauer; individualCount: 1; sex: male; lifeStage: adult; occurrenceID: 3C592CCE-FA55-5E3E-8356-78973CAA1C8A; **Taxon:** scientificName: Bradysia polonica; **Location:** country: Germany; stateProvince: Baden-Württemberg; locality: Backnang, Katharinenplaisir, Streuobstwiese; verbatimElevation: 294 m; verbatimCoordinates: 48°57'19.34"N 9°26'25.82"E; **Identification:** identifiedBy: Roy J. Canty & Hans-Georg Rudzinski; **Event:** samplingProtocol: Malaise Trap; eventDate: 28/V–16/VI/2014; **Record Level:** institutionID: SMNS
- **Type status:** Other material. **Occurrence:** catalogNumber: SMNS_DIP_022583; recordedBy: A. Rosenbauer; individualCount: 1; sex: male; lifeStage: adult; occurrenceID: 6992FCA4-FCD0-5A82-825A-A2697FC4BBC0; **Taxon:** scientificName: Bradysia polonica; **Location:** country: Germany; stateProvince: Baden-Württemberg; locality: Backnang, Katharinenplaisir, Streuobstwiese; verbatimElevation: 294 m; verbatimCoordinates: 48°57'19.34"N 9°26'25.82"E; **Identification:** identifiedBy: Roy J. Canty & Hans-Georg Rudzinski; **Event:** samplingProtocol: Malaise Trap; eventDate: 28/V–16/VI/2014; **Record Level:** institutionID: SMNS
- **Type status:** Other material. **Occurrence:** catalogNumber: SMNS_DIP_022620; recordedBy: A. Rosenbauer; individualCount: 1; sex: male; lifeStage: adult; occurrenceID: FC57633B-DE33-500C-8B54-BBDE0963DDF1; **Taxon:** scientificName: Bradysia polonica; **Location:** country: Germany; stateProvince: Baden-Württemberg; locality: Backnang, Katharinenplaisir, Streuobstwiese; verbatimElevation: 294 m; verbatimCoordinates: 48°57'19.34"N 9°26'25.82"E; **Identification:** identifiedBy: Roy J. Canty & Hans-Georg Rudzinski; **Event:** samplingProtocol: Malaise Trap; eventDate: 28/V–16/VI/2014; **Record Level:** institutionID: SMNS
- **Type status:** Other material. **Occurrence:** catalogNumber: SMNS_DIP_022669; recordedBy: A. Rosenbauer; individualCount: 1; sex: male; lifeStage: adult; occurrenceID: 0A50D9A4-2BBC-5712-BB72-01CBBDE671B4; **Taxon:** scientificName: Bradysia polonica; **Location:** country: Germany; stateProvince: Baden-Württemberg; locality: Backnang, Katharinenplaisir, Streuobstwiese; verbatimElevation: 294 m; verbatimCoordinates: 48°57'19.34"N 9°26'25.82"E; **Identification:** identifiedBy: Roy J. Canty & Hans-Georg Rudzinski; **Event:** samplingProtocol: Malaise Trap; eventDate: 28/V–16/VI/2014; **Record Level:** institutionID: SMNS
- **Type status:** Other material. **Occurrence:** catalogNumber: SMNS_DIP_023326; recordedBy: T. Kothe, M. Engelhardt, Ch. Köking; individualCount: 1; sex: male; lifeStage: adult; occurrenceID: 96FBB99C-C524-5228-A1FA-DC85620B335D; **Taxon:** scientificName: Bradysia polonica; **Location:** country: Germany; stateProvince: Baden-Württemberg; locality: Tübingen, Steinenberg; verbatimElevation: 492m; verbatimCoordinates: 48°31'52"N 9°01'48"E; **Identification:** identifiedBy: Roy J. Canty & Hans-Georg Rudzinski; **Event:** samplingProtocol: Malaise Trap; eventDate: 25/IV–13/V/2014; **Record Level:** institutionID: SMNS
- **Type status:** Other material. **Occurrence:** catalogNumber: SMNS_DIP_023332; recordedBy: T. Kothe, M. Engelhardt, Ch. Köking; individualCount: 1; sex: male; lifeStage: adult; occurrenceID: 1B06CDFF-EED2-5036-8287-ECDD83812539; **Taxon:** scientificName: Bradysia polonica; **Location:** country: Germany; stateProvince: Baden-Württemberg; locality: Tübingen, Steinenberg; verbatimElevation: 492m; verbatimCoordinates: 48°31'52"N 9°01'48"E; **Identification:** identifiedBy: Roy J. Canty & Hans-Georg Rudzinski; **Event:** samplingProtocol: Malaise Trap; eventDate: 25/IV–13/V/2014; **Record Level:** institutionID: SMNS
- **Type status:** Other material. **Occurrence:** catalogNumber: SMNS_DIP_024446; recordedBy: T. Kothe, M. Engelhardt, Ch. Köking; individualCount: 1; sex: male; lifeStage: adult; occurrenceID: 722AC5D7-6FDF-5E39-AD4C-A7125D57C3FE; **Taxon:** scientificName: Bradysia polonica; **Location:** country: Germany; stateProvince: Baden-Württemberg; locality: Tübingen, Steinenberg; verbatimElevation: 492m; verbatimCoordinates: 48°31'52"N 9°01'48"E; **Identification:** identifiedBy: Roy J. Canty & Hans-Georg Rudzinski; **Event:** samplingProtocol: Malaise Trap; eventDate: 25/IV–13/V/2014; **Record Level:** institutionID: SMNS
- **Type status:** Other material. **Occurrence:** catalogNumber: SMNS_DIP_024499; recordedBy: T. Kothe, M. Engelhardt, Ch. Köking; individualCount: 1; sex: male; lifeStage: adult; occurrenceID: EBBBD536-A055-5815-AA1A-84F9FFF22D41; **Taxon:** scientificName: Bradysia polonica; **Location:** country: Germany; stateProvince: Baden-Württemberg; locality: Tübingen, Steinenberg; verbatimElevation: 492m; verbatimCoordinates: 48°31'52"N 9°01'48"E; **Identification:** identifiedBy: Roy J. Canty & Hans-Georg Rudzinski; **Event:** samplingProtocol: Malaise Trap; eventDate: 25/IV–13/V/2014; **Record Level:** institutionID: SMNS
- **Type status:** Other material. **Occurrence:** catalogNumber: ZFMK-TIS-2531984; recordedBy: K. Heller; individualCount: 1; sex: male; lifeStage: adult; occurrenceID: 7FDE538E-5C4F-5B42-BD8A-76645D164394; **Taxon:** scientificName: Bradysia polonica; **Location:** country: Germany; stateProvince: Hamburg; locality: Hamburg-Farmsen, Kupferteich; verbatimCoordinates: 53°34'59.999988"N 10°7'0.000012"E; **Identification:** identifiedBy: K. Heller; **Event:** samplingProtocol: Sweep net; eventDate: 25/VIII/2014; **Record Level:** institutionID: ZFMK
- **Type status:** Other material. **Occurrence:** catalogNumber: ZFMK-TIS-2605572; recordedBy: K. Heller; individualCount: 1; sex: male; lifeStage: adult; occurrenceID: 740725C0-4398-5BA0-97CA-2501CA7E90B4; **Taxon:** scientificName: Bradysia polonica; **Location:** country: Germany; stateProvince: Sachsen-Anhalt; locality: NSG Selketal; verbatimCoordinates: 51°40'14.88"N 11°10'18.12"E; **Identification:** identifiedBy: K. Heller; **Event:** samplingProtocol: Sweep net; eventDate: 29/VII/2017; **Record Level:** institutionID: ZFMK
- **Type status:** Other material. **Occurrence:** catalogNumber: ZFMK-TIS-2605573; recordedBy: K. Heller; individualCount: 1; sex: male; lifeStage: adult; occurrenceID: EAC248FA-425E-5625-B3A1-50268DF6512F; **Taxon:** scientificName: Bradysia polonica; **Location:** country: Germany; stateProvince: Sachsen-Anhalt; locality: NSG Selketal; verbatimCoordinates: 51°40'14.88"N 11°10'18.12"E; **Identification:** identifiedBy: K. Heller; **Event:** samplingProtocol: Sweep net; eventDate: 29/VII/2017; **Record Level:** institutionID: ZFMK
- **Type status:** Other material. **Occurrence:** catalogNumber: ZFMK-TIS-2605577; recordedBy: K. Heller; individualCount: 1; sex: male; lifeStage: adult; occurrenceID: 2683EA08-175D-51D0-A720-8C4A7C3516E7; **Taxon:** scientificName: Bradysia polonica; **Location:** country: Germany; stateProvince: Sachsen-Anhalt; locality: NSG Selketal; verbatimCoordinates: 51°40'14.88"N 11°10'18.12"E; **Identification:** identifiedBy: K. Heller; **Event:** samplingProtocol: Sweep net; eventDate: 29/VII/2017; **Record Level:** institutionID: ZFMK
- **Type status:** Other material. **Occurrence:** catalogNumber: ZFMK-TIS-2605610; recordedBy: P. Grootaert; individualCount: 1; sex: male; lifeStage: adult; occurrenceID: 80494886-6BA8-52E5-B0C8-3D4256D365C6; **Taxon:** scientificName: Bradysia polonica; **Location:** country: Belgium; stateProvince: Brussels; locality: Jean Massart Botanical Garden; verbatimCoordinates: 50°48'51.9984"N 4°26'17.9988"E; **Identification:** identifiedBy: K. Heller; **Event:** samplingProtocol: Malaise Trap; eventDate: 09/VI/2016; **Record Level:** institutionID: ZFMK

#### Diagnosis

**Figure 1.**
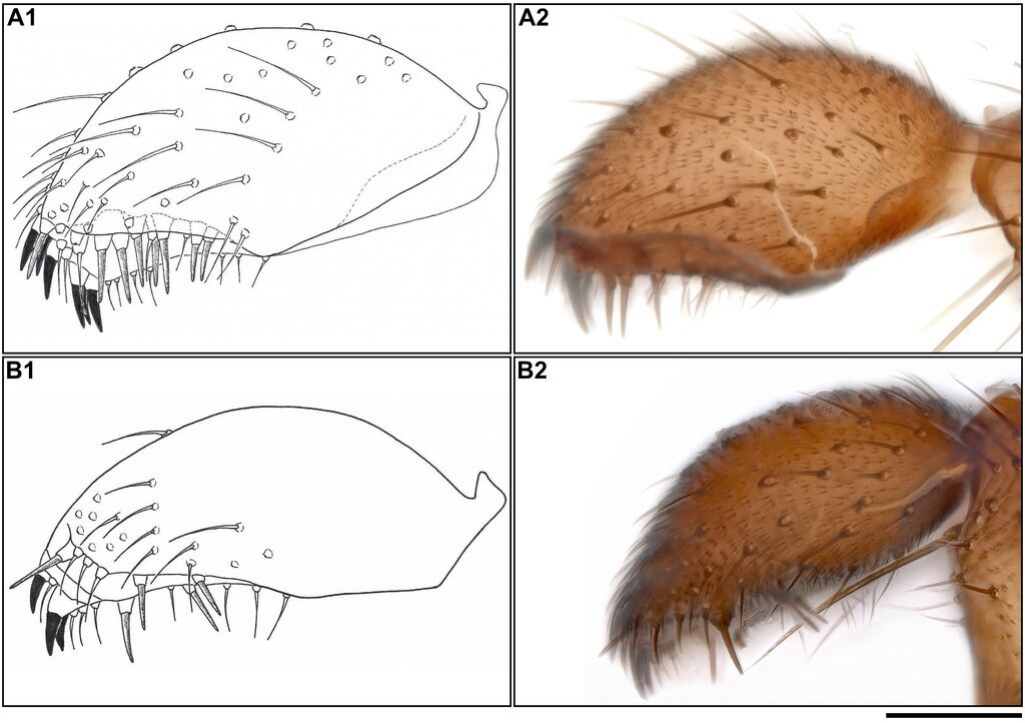
*Bradysia polonica*: (A1) hand-drawn dorsal view of the gonostylus, and (A2) photographed gonostylus; *Bradysia spinidensa*: (B1) hand-drawn dorsal view of the gonostylus, and (B2) photographed gonostylus.

All based on the specimens observed (34 specimens). The gonostylus (Fig. 1 A1 & A2) with: 3–7 claw-like spines found apically-subapically on the inside; 6–10 hyaline spines (1 subapical, 5–9 spines inside the inner groove, and randomly clustered). L/mB index of gonostylus: 1.73–2.23.

In comparison to *B. spinidensa* stat. res., the only discernible differences that could be observed were found in the gonostylus. Even though the gonostylus of *B. polonica* tends to be thicker (with a generally lower L/mB index), and with more claw-like spines on the apex-subapex, there is some overlap. The most consistent and reliable difference is in the number and formation of the hyaline spines. *B. polonica* has more hyaline spines (6–10 in *B. polonica* versus 4–5 in *B. spinidensa*), which are arranged randomly within the inner groove, compared to *B. spinidensa* where the hyaline spines are arranged in pairs.

#### Taxon discussion

Menzel et al. (1990) synonymised *B. edwardsi* with *B. polonica* without comment. Our observations on the original description and illustrations of *B. edwardsi* by Freeman (1983), combined with discussions with Björn Rulik and Kai Heller, and observations of images of the holotype from the Natural History Museum, London’s online database (Natural History Museum, London 2024), agree with this synonymy.

### Bradysia spinidensa

Hondru, 1968

E98ACFD3-24F0-511D-B26E-87B6A0957DA3

Hondru 1968 Rev. roum. bioI. (Zool.), 13(2): 92-94; Fig. 4 A-D.
Type Locality: Copăceni and Voluntaru (Bezirk Ilfov, Romania).
Syntypes: 1 male 27.IX.1962 (Copăceni); 6 males, 2.VIII.1964 (Copăceni); 7 males, 5.IX.–15.IX.1964 (Copăceni); 3 males, 18.IV.1962 (Voluntaru); all leg. HONDRU.

#### Materials

- **Type status:** Other material. **Occurrence:** catalogNumber: SMNS_DIP_022418; recordedBy: A. Rosenbauer; individualCount: 1; sex: male; lifeStage: adult; occurrenceID: 7107FC9B-2E75-5A97-9485-2607DA1BB442; **Taxon:** scientificName: Bradysia spinidensa; **Location:** country: Germany; stateProvince: Baden-Württemberg; locality: Backnang, Katharinenplaisir, Streuobstwiese; verbatimElevation: 294 m; verbatimCoordinates: 48°57'19.34"N 9°26'25.82"E; **Identification:** identifiedBy: Roy J. Canty & Hans-Georg Rudzinski; **Event:** samplingProtocol: Malaise Trap; eventDate: 28/V–16/VI/2014; **Record Level:** ownerInstitutionCode: SMNS
- **Type status:** Other material. **Occurrence:** catalogNumber: SMNS_DIP_022646; recordedBy: A. Rosenbauer; individualCount: 1; sex: male; lifeStage: adult; occurrenceID: CBBFD3AE-63FB-5B64-9F91-D77E6666F2FF; **Taxon:** scientificName: Bradysia spinidensa; **Location:** country: Germany; stateProvince: Baden-Württemberg; locality: Backnang, Katharinenplaisir, Streuobstwiese; verbatimElevation: 294 m; verbatimCoordinates: 48°57'19.34"N 9°26'25.82"E; **Identification:** identifiedBy: Roy J. Canty & Hans-Georg Rudzinski; **Event:** samplingProtocol: Malaise Trap; eventDate: 28/V–16/VI/2014; **Record Level:** ownerInstitutionCode: SMNS
- **Type status:** Other material. **Occurrence:** catalogNumber: SMNS_DIP_029186; recordedBy: HTW-Dresden/SMNS; individualCount: 1; sex: male; lifeStage: adult; occurrenceID: ED80888B-0952-5C8D-AF9A-E7F2E7D28E99; **Taxon:** scientificName: Bradysia spinidensa; **Location:** country: Germany; stateProvince: Sachsen; locality: Lkr. Sächsische Schweiz-Osterzgebirge, Bad Gottleuba-Berggießhübel, NSG-Mittelgebirgslandschaft um Oelsen, Sattelbergwiese; verbatimElevation: 607 m; verbatimCoordinates: 50°47'24.18"N 13°55'50.2212"E; **Identification:** identifiedBy: Roy J. Canty & Hans-Georg Rudzinski; **Event:** samplingProtocol: Malaise Trap; eventDate: 03/V–16/VI/2014; **Record Level:** ownerInstitutionCode: SMNS
- **Type status:** Other material. **Occurrence:** catalogNumber: SMNS_DIP_029207; recordedBy: HTW-Dresden/SMNS; individualCount: 1; sex: male; lifeStage: adult; occurrenceID: D8138DAC-A30C-56F3-8351-DB137AD85B06; **Taxon:** scientificName: Bradysia spinidensa; **Location:** country: Germany; stateProvince: Sachsen; locality: Lkr. Sächsische Schweiz-Osterzgebirge, Bad Gottleuba-Berggießhübel, NSG-Mittelgebirgslandschaft um Oelsen, Sattelbergwiese; verbatimElevation: 607 m; verbatimCoordinates: 50°47'24.18"N 13°55'50.2212"E; **Identification:** identifiedBy: Roy J. Canty & Hans-Georg Rudzinski; **Event:** samplingProtocol: Malaise Trap; eventDate: 03/V–16/VI/2014; **Record Level:** ownerInstitutionCode: SMNS
- **Type status:** Other material. **Occurrence:** catalogNumber: ZFMK-TIS-2583290; recordedBy: M. Jaschhof, C. Jaschhof, M. Karström; individualCount: 1; sex: male; lifeStage: adult; occurrenceID: EA3F5E0C-5C6A-5F03-9297-8679CF048B5E; **Taxon:** scientificName: Bradysia edwardsi; **Location:** country: Sweden; locality: Jokkmokk, Vuollerim; verbatimCoordinates: 66°25'47.830"N 20°37'27.771"E; **Identification:** identifiedBy: K. Heller; **Event:** samplingProtocol: Malaise Trap; eventDate: 17/XI/2016; **Record Level:** ownerInstitutionCode: ZFMK
- **Type status:** Other material. **Occurrence:** catalogNumber: ZFMK-TIS-2583313; recordedBy: M. Jaschhof, C. Jaschhof, M. Karström; individualCount: 1; sex: male; lifeStage: adult; occurrenceID: 3C810A48-2FB5-52A8-BE3B-D0833D134321; **Taxon:** scientificName: Bradysia edwardsi; **Location:** country: Sweden; locality: Jokkmokk, Vuollerim; verbatimCoordinates: 66°25'47.830"N 20°37'27.771"E; **Identification:** identifiedBy: K. Heller; **Event:** samplingProtocol: Malaise Trap; eventDate: 17/XI/2016; **Record Level:** ownerInstitutionCode: ZFMK

#### Diagnosis

All based on specimens observed (6 specimens). The gonostylus (Fig. 1 B1 & B2) with: 3–4 claw-like spines found apically-subapically on the inside; 4–5 hyaline spines (1 subapical, 3–4 spines inside the inner groove, typically paired). L/mB index of the gonostylus is in the range of 2.11–2.625.

In comparison with *B. polonica*, the gonostylus tends to be thinner (with a higher L/mB index), and with fewer claw-like spines on the apex-subapex, albeit with some overlap. The main consistent difference between *B. spinidensa* and *B. polonica* is the number and formation of the hyaline spines. *B. spinidensa* has 4–5 hyaline spines which are arranged in pairs inside the inner groove, compared to the 6–10 hzaline spines in *B. polonica*, which are arranged randomly inside the inner groove.

#### Taxon discussion

While the differences might appear to be minor, the fact that these differences are consistent and congruent with our molecular groups strongly suggests that these are two separate species. As stated in our introduction, *Bradysia edwardsi* is clearly congruent with *Bradysia polonica*; however, the same level of certainty is not available regarding *Bradysia spinidensa*.

When Menzel and Mohrig (2000) synonymised *B. spinidensa* with *B. polonica*, they did so without explaining their reasoning and without having observed the type material of *B. spinidensa*, as they could not confirm its location due to the lack of response to their enquiries with Hondru’s place of work (unnamed), which they attributed to the political and financial situation in Romania at the time (Menzel and Mohrig 2000). Our own enquiries with the Grigore Antipa National Museum of Natural History, Bucharest; the Natural History Museum, Faculty of Biology, University Alexandru Ioan Cuza in Iasi; and the Institute of Biology Bucharest of the Romanian Academy found that there are no specimens of *B. spinidensa* or other specimens identified by Hondru in their collections. Enquiries into what turned out to be Hondru’s last place of work, the Research Development Institute for Plant Protection, Bucharest (ICDPP), also did not lead to finding the type material. Despite the impossibility to study the type material, we consider the original description as sufficiently informative to question Menzel and Mohrig's synonymy.

The original description of *B. spinidensa* by Hondru (1968) is very minimal, with very little detail provided. However, the drawings of the hypopygium and gonostyli provided by Hondru (1968; fig. 4 A and B, page 93) do appear to resemble the hypopygium and gonostyli of our specimens in both size and shape, especially with the more elongate gonostyli compared to those of *B. polonica*. Additionally, Hondru (1968) described the gonostylus of *B. spinidensa* as having two strong spines on the inner-side of the gonostylus (the thicker, darker spines): one apically, the other subapically and inside the inner groove. While this is one or two fewer spines than what we observed, it is possible that the specimens studied by Hondru were damaged, that spines were overlooked, or that they genuinely had one or two fewer than stated in the description.

It could also be that the specimens here identified as *B. spinidensa* potentially represent a so-far undescribed species that shows only minimal divergence from the Romanian species *B. spinidensa*. However, describing this taxon as new, without being completely sure, would not contribute to a more stable taxonomy. With no material available from the type locality of *B. spinidensa*, which would allow for direct comparisons, and with the type specimens still missing, we cannot be completely certain that our specimens represent a new species. We therefore refrain from describing it as a separate species at this time. The German specimens show morphological similarities to *B. spinidensa*, with only minor differences in the gonostyli the origin and taxonomic significance of which remain uncertain.

For the above reasons and despite the holotype of *B. spinidensa* being probably lost, we also cannot designate a neotype for that nominal species. None of our specimens were collected from anywhere near the type locality, which would not satisfy article 75.3.6 of the ICZN (1999). The mentioned uncertainty regarding the conspecificity of our specimens with *B. spinidensa* would also not satisfy article 75.3.5 of the ICZN (1999).

## Analysis

### Molecular

**Figure 2.**
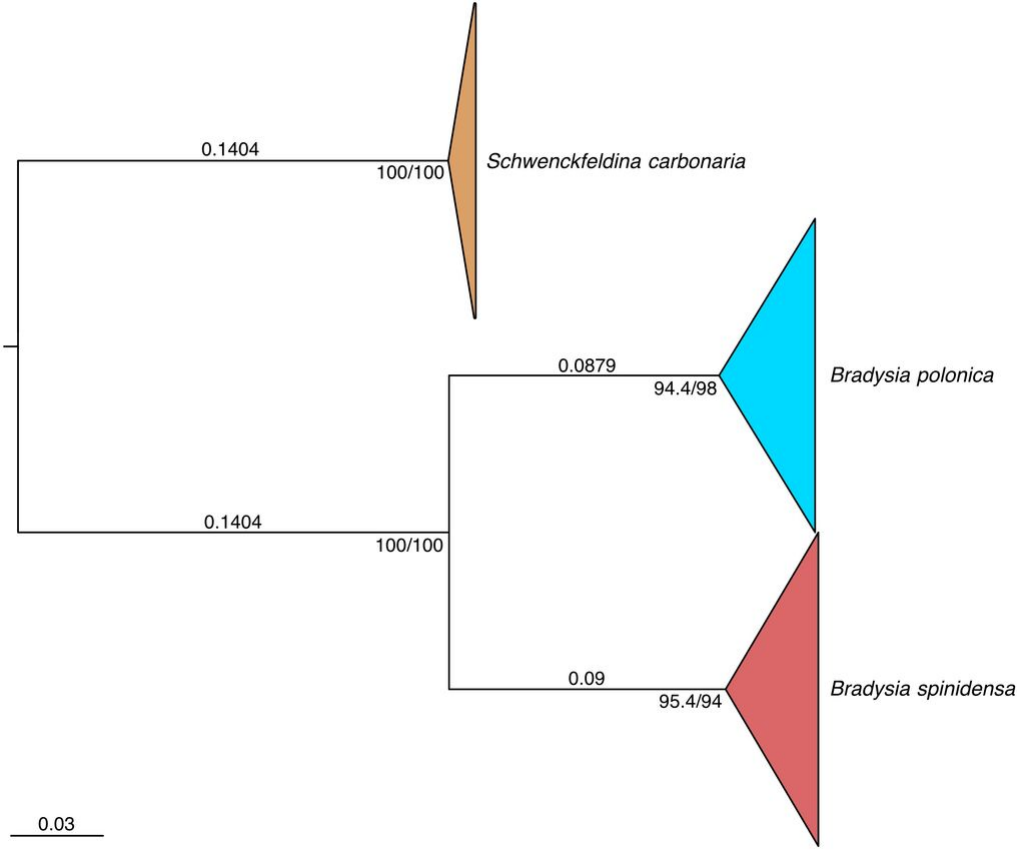
COI phylogenetic tree of *Bradysia polonica* and *Bradysia spinidensa* with the *Schwenckfeldina carbonaria* outgroup. Numbers at the branch tips indicate percentage support values (SH-aLRT/Ultrafast bootstrap). Numbers on the branches indicate substitution rate.

The ASAP analysis hypothesized from 2 to 16 potential molecular species clusters (Table 1). The best scoring (lowest ASAP-score), and therefore the most likely (according to ASAP), hypothesis indicates two species groups with a threshold distance of 0.065. The phylogenetic tree produced by IQ-Tree (as per the materials and methods section) also shows a clear separation between two distinct molecular clusters (Fig. 2; Suppl. material 1), sharing the same specimens in these clusters with the best scoring ASAP results (Suppl. material 2). Both clusters show strong SH–aLRT (94.4% for *B. polonica*-group; 95.4% for *B. spinidensa*-group) and Ultrafast bootstrap support (98% for *B. polonica*-group and 94% for *B. spinidensa*-group).

### Morphology

Specimens within both molecular clusters showed morphological variation in the gonostylus regarding the placement and number of the two sets of spines (the thicker, darker spines, and thinner, hyaline spines) and the L/mB index, sometimes even within the same specimen. However, while there was some morphological overlap between the two clusters, there were also some consistent morphological differences (Suppl. materials 3, 4). Specimens in both clusters have a groove that runs on the inside of the gonostylus from the apex, varying in length. Additionally, there are two sets of spines on the gonostylus: thicker, claw-like spines found apically to subapically (organised in pairs; if odd-numbered, then one lone spine), and thinner, hyaline spines: 1 subapical, among the claw-like spines, the rest located further back, between the apex and the middle of the gonostylus, within the above-mentioned groove.

## Discussion

The use of an integrative method combining molecular barcode data with a subsequent morphological analysis has often shown effectiveness in finding previously overlooked morphological characters to aid in distinguishing sciarid species, as well as species discovery, even when there are minor morphological differences (Heller et al. 2016). This certainly appears to be the case in this study, with the seemingly minor, but consistent differences in the gonostyli between *B. polonica* and *B. spinidensa*: *B. polonica* with: an L/mB index of 1.73–2.23; 3–7 claw-like spines found apically-subapically on the inside; and 6–10 hyaline spines arranged thusly: 1 subapical, 5–9 spines inside the inner groove, and randomly clustered; and *B. spinidensa* with an L/mB index of the gonostylus is in the range of 2.11–2.625; 3–4 claw-like spines found apically-subapically on the inside; and 4–5 hyaline spines arranged thusly: 1 subapical, 3–4 spines inside the inner groove, paired. This study has therefore effectively utilised such a methodology with molecular barcode data and morphology to uncover and separate *Bradysia spinidensa* from *Bradysia polonica*.

Additionally, a further study that will utilise this integrative taxonomic method as part of this sub-project on Sciaridae for GBOL III is also in preparation. This study will focus on two species groups well known to have cryptic diversity: *Bradysia trivittata* (Staeger, 1840), and *Corynoptera tridentata* Hondru, 1968 (Kolcsár and Heller 2019).

## Supplementary Material

XML Treatment for Bradysia polonica

XML Treatment for Bradysia polonica

XML Treatment for Bradysia spinidensa

EF0C3270-E119-5742-B03E-ED1D74220DF1 10.3897/BDJ.13.e171689.suppl1 Supplementary material 1 Supplementary tree 1 Data type Phylogenetic Tree as a NEXUS File Brief description Phylogenetic tree output from the IQ Tree analysis of *Bradysia polonica* and *Bradysia spinidensa* with *Schwenckfeldina carbonaria* as the outgroup. Numbers at the branch tips indicate percentage support values (SH-aLRT/Ultrafast bootstrap). Numbers on the branches indicate substitution rate. File: oo_1381111.newick https://binary.pensoft.net/file/1381111 Roy J Canty, Hans-Georg Rudzinski, Dominic Wanke, Daniel Whitmore

D3FC1D1D-B5B8-5810-969A-A8E04AFBDF5D 10.3897/BDJ.13.e171689.suppl2 Supplementary material 2 Supplementary tree 2 Data type ASAP cluster data svg file Brief description Phylogenetic output from the ASAP analysis showing the most likely molecular species clusters, along with Nb subsets/asap scores and rank at the top of the tree. File: oo_1381149.svg https://binary.pensoft.net/file/1381149 Roy J Canty, Hans-Georg Rudzinski, Dominic Wanke, Daniel Whitmore

30AEAA5F-9C62-586D-BE14-D01EBA2E990B 10.3897/BDJ.13.e171689.suppl3 Supplementary material 3 Supplementary Table 1 Data type Excel Spreadsheet Brief description Table displaying the key observed morphometric data of the gonostyli of *Bradysia polonica*. File: oo_1479635.xlsx https://binary.pensoft.net/file/1479635 Roy J Canty, Hans-Georg Rudzinski, Dominic Wanke, Daniel Whitmore

4D5106EA-EBB3-5F98-A04F-68117091DD8F 10.3897/BDJ.13.e171689.suppl4 Supplementary material 4 Supplementary Table 2 Data type Excel Spreadshee Brief description Table displaying the key observed morphometric data of the gonostyli of *Bradysia spinidensa*. File: oo_1479637.xlsx https://binary.pensoft.net/file/1479637 Roy J Canty, Hans-Georg Rudzinski, Dominic Wanke, Daniel Whitmore

## Figures and Tables

**Table 1.** ASAP results showing the number of potential molecular species (Nb of subsets), ranked by the ASAP score, which is based on the rankings of both the P-value rank and the relative gap width (W). The lower the ASAP score, the better the probability.

Nb of subsets	ASAP-score	P-val (rank)	W (rank)	Threshold dist.
2	1.00	1.00e-05 (1)	5.41e-05 (1)	0.064985
5	3.50	1.96e-01 (4)	1.20e-05 (3)	0.010735
7	4.00	3.06e-02 (2)	7.73e-07 (6)	0.001615
3	4.00	8.94e-01 (6)	3.58e-05 (2)	0.023556
6	5.00	4.91e-01 (5)	9.67e-07 (5)	0.001711
16	5.50	8.96e-02 (3)	5.87e-08 (8)	0.000760
4	5.50	1.00e+00 (7)	1.07e-05 (4)	0.020517
